# Profiling of microorganism-binding serum antibody specificities in professional athletes

**DOI:** 10.1371/journal.pone.0203665

**Published:** 2018-09-25

**Authors:** Rajna Minić, Zlatko Papić, Brižita Đorđević, Danica Michaličkova, Vesna Ilić, Geir Mathiesen, Irena Živković, Visnja Pantic, Ljiljana Dimitrijević

**Affiliations:** 1 Department of Scientific Research, Institute of Virology, Vaccines and Sera, Torlak, Belgrade, Serbia; 2 University of Leeds, School of Physics and Astronomy, University of Leeds, E C Stoner Building, Leeds, United Kingdom; 3 Department of Bromatology, Faculty of Pharmacy, University of Belgrade, Belgrade, Serbia; 4 Institute of Pharmacology, First Faculty of Medicine, Charles University, Prague, Czech Republic; 5 Institute for Medical Research, University of Belgrade, Belgrade, Serbia; 6 Department of Chemistry, Biotechnology and Food Science, Norwegian University of Life Sciences (NMBU), Ås, Norway; Laurentian, CANADA

## Abstract

The goal of this work was to elucidate similarities between microorganisms from the perspective of the humoral immune system reactivity in professional athletes. The reactivity of serum IgG of 14 young, individuals was analyzed to 23 selected microorganisms as antigens by use of the in house ELISA. Serum IgM and IgA reactivity was also analyzed and a control group of sex and age matched individuals was used for comparison. The obtained absorbance levels were used as a string of values to correlate the reactivity to different microorganisms. IgM was found to be the most cross reactive antibody class, Pearson’s *r* = 0.7–0.92, for very distant bacterial species such as *Lactobacillus* and *E*. *coli*.High correlation in IgG levels was found for Gammaproteobacteria and LPS (from *E*. *coli*) (*r =* 0.77 for LPS vs. *P*. *aeruginosa* to *r =* 0.98 for LPS vs. *E*.*coli*), whereas this correlation was lower in the control group (*r =* 0.49 for LPS vs. *P*. *aeruginosa* to *r =* 0.66 for LPS vs. *E*.*coli*). The correlation was also analyzed between total IgG and IgG subclasses specific for the same microorganism, and IgG2 was identified as the main subclass recognising different microorganisms, as well as recognising LPS. Upon correlation of IgG with IgA for the same microorganism absence of or negative correlation was found between bacteria-specific IgA and IgG in case of *Lactobacillus* and *Staphylococcus*geni, whereas correlation was absent or positive for *Candida albicans*, *Enterococcusfaecalis*,*Streptococcus* species tested in professional athletes. Opposite results were obtained for the control group. Outlined here is a simple experimental procedure and data analysis which yields functional significance and which can be used for determining the similarities between microorganisms from the aspect of the humoral immune system, for determining the main IgG subclass involved in an immune response as well as for the analysis of different target populations.

## Introduction

Microorganisms inhabit human body surfaces like the skin and mucosal tissues and thereby directly influence our lives. There are various methods for classifying and identifying microorganisms based on morphological characteristics, differential staining, biochemical testing, selective cultivation and gene sequencing [1,2].

For example the analysis of microbial DNA at body habitats such as the gut, the oral cavity, nostrils, and different skin sites revealed that most sequences belonged to the phyla *Firmicutes*, *Bacteroidetes*, *Actinobacteria* and *Proteobacteria*, that each body habitat has a unique composition of microbiota which is relatively stable over time, but varies between individuals [3,4,5]. The stability of microbiota at different sites within an individual implies a mutually beneficial stable coexistence with the human host, and the disturbance of microbiota might provide an indication of a disease [6].Besides the identification of microorganisms at different body habitats it is of importance to have an understanding of the nature of this mutualism/commensalism. The reactivity of serum antibodies toward different bacteria shapes the relationship, at least on mucosal surfaces [7].

Several reviews have been published recently on the comeback of monoclonal antibacterial antibodies as passive treatment of bacterial infections [8,9]. In the present study specificity of serum polyclonal antibodies was used to explore the similarities between microorganisms with which humans get in contact with, by measuring serum antibody levels to microorganisms. This is a very important aspect of host-microorganism interaction, as it serves as an indication of the level of microbial burden in a given population and might indicate predisposition to certain infections or other inflammatory diseases. The populationsanalyzed in the present study consisted of young, healthy athletes and the tested microorganisms were selected as relevant in epidemiology and daily life. The microorganisms species tested are all relevant as being in close contact with humans, *Streptococcus* species and *Staphylococcus* as upper respiratory tract pathogens, Lactobacilli were tested as bacteria which are often taken as oral probiotic supplements, whereas Gammaproteobacteria were tested as relevant opportunistic pathogens, which posses LPS, the prototypical endotoxin widely used in biological experimentation.Professional athletes were chosen due to absence of pathology and relative population uniformity, but they at the same time represent an immunologically specific population in a sense that prolonged vigorous exercise has a negative impact on the immune system, which leads to an increased infection rate (upper respiratory tract infections) [10] and duration. Strenuous exercise induces high core body temperature and reduction in splanchnic blood flow which leads to the disruption of tight junctions, the increase of GI barrier permeability, bacterial translocation and higher levels of circulating LPS [11],which makes the selected study population unique.

The goal of the present work is to analyze the antibody reactivity of whole sera to microorganisms in professional athletes, and to see what are the correlation levels towards different microorganisms across the population, as well as to test different antibody classes and subclasses to determine mutual relationships.

## Materials and methods

### Study subjects

Athletes (n = 14) included in the study were professionals training in triathlon, cycling, alpinism, savate box and swimming. Physical and anthropometric characteristics of the participants were: 10 men (maximal oxygen uptake (VO_2max_) = 49–82 mL/(kg•min)) and 4 women (VO_2max_ = 45–50 mL/(kg•min)), BMI:21.2–23.6 kg/m2 for men and 20.9–24.5 kg/m2 for women, % fat:7.3–11.9% for men; 17.8–20.5% for women, aged 18–28 years, nonsmokers, with high training load >11 h/week [12]. As a control group 14 individuals (10 men, 4 women), 19–26 years old, none professional athletes, were used. Exclusion criteria were the use of probiotics and antibiotics a month before the start sample collection, recent surgical intervention, and the presence of chronic diseases.

Experiment was in accordance with The Code of Ethics of the World Medical Association (Declaration of Helsinki) and approved by the Ethics Committee of Sports Medicine Association of Serbia.The subjects signed an informed consent.

Blood samples were collected between 9:30 and 10:30 AM; serum was separated by centrifugation at 1 500 x g, 15 min, and stored at -20° C until analysis.

### Bacterial strains, growth conditions

Bacterial and fungal strains used in this study are listed in Table 1. *Lactobacillus* species were propagated in MRS broth (“Torlak”, Serbia) at 37°C, the other bacterial species were grown in BHI broth (BD, USA) at 37°C. *Candida albicans* was grown in Sabouraud dextrose broth (SDB) at 25°C.

**Table 1:** Microorganism used in present study.

Microorganism	Phylum	Genus
*Lactobacillus reuteri* DSM 17938	Firmicutes G+	Lactobacillus
*Lactobacillus plantarum* WCFS1		
*Lactobacillus rhamnosus* LA68		
*Lactobacillus rhamnosus* LB64		
*Lactobacillus rhamnosus* LGG		
*Lactobacillus helveticus* LAFTI L10		
*Lactobacillus acidophilus*ViVag		
*Lactobacillus casei* DG		
*Streptococcus pyogenes* ATCC 19615		Streptococcus
*Streptococcus agalactiae* ATCC 13813		
*Streptococcus sp*. CI β-hemolytic group B		
*Streptococcus* sp. CI β-hemolytic group A		
*Enterococcus faecalis* CI*	Proteobacteria G-	Enterococcus
*Staphylococcus aureus* CI		Staphylococcus
*Proteus mirabilis* CI		Proteus
*Escherichia coli* CI		Escherichia
*Salmonella typhimurium* 2865		Salmonella
*Klebsiella pneumoniae* ATCC 13883		Klebsiella
*Pseudomonas aeruginosa* ATCC 27853		Pseudomonas
*Proteus hauseri* ATCC 13315		Proteus
*Shigella flexneri* ATCC 12022		Shigella
*Escherichia coli* ATCC25922		Escherichia
*Candida albicans* ATCC 10259	Ascomycota	Candida

^*^ CI- clinical isolate

Overnight cultureswere centrifuged (2000 x g, 20 min, 4°C), washed once and diluted in PBS to optical density of 0.1 at 610 nm in a final volume of 200 μL in 96 well plate. After applying 50 μl per wellto MaxiSorp ELISA plates (Nunc, ThermoFisher Scientific, Denmark), plates were centrifuged (1000 x g, 20 min, 24°C) and supernatant was removed. The plates were dried at 50°C for two hours, and stored for no longer than 10 days prior to usage.

### Anti-bacterial ELISA

The procedure was as previously described [13]. The plates were blocked with 2% bovine serum albumin (BSA)/PBS at room temperature (RT) for 1 h, and then washed 3 times with PBS. For the analysis of bacteria-specific IgG, the sera were diluted 400, and for specific IgA and IgM sera were diluted 100 times in 1% BSA/PBS. For the analysis of *S*. *aureus* CI specific (IgG and IgA) antibodies preincubation was done with non-immunized rabbit sera, at a dilution 1:10, for 1h at RT. Sera was incubated for 2 hours at RT and then washed 3 times with PBS. The following secondary antibodies were used: monoclonal anti-human IgG (Fc specific) biotin conjugate, anti-human IgM (μ-chain specific) biotin conjugate, anti-human IgA (α-chain specific) (Sigma Aldrich, St. Louis, MO, USA). For IgG subclass analysis, Biotin Mouse Anti-Human IgG1, IgG2, IgG4 (BD Biosciences, CA, USA), and Monoclonal Anti-Human IgG3-Biotin antibody produced in mouse (Sigma Aldrich) were used.All secondary antibodies were incubated for 1 hour at RT. After washing with PBS, streptavidin–horse radish peroxidase (Biolegend, San Diego, CA, USA) was added and incubated for 1 hour at RT. SigmaFast OPD (Sigma Aldrich) was used as substrate. The absorbance was read at 492 nm with reference wavelength at 620 nm. Each sample was measured in duplicate, and intra-assay coefficients of variation were below or equal to 10% for all the bacterial ELISAs performed.

### Anti-LPS ELISA

Antibodies specific for LPS were tested with the following procedure, briefly 50 μl LPS (25 μg/mL) from *E*. *coli* (O55:B5, Difco Laboratories) was adsorbed onto microtiter plate (MaxiSorp, Nunc), first for 2 h at 37°C, and then overnight at 4°C. The plate was then blocked with 1% BSA/PBS (2 h, RT), and washed with PBS. Anti-LPS IgG was detected at a dilution of 400 x, and IgA at a dilution 100 x.

### Statistical analysis

Pearson product-moment correlation coefficients (*r*) were calculated using GraphPad Prismfor the levels of IgM, IgG and IgA specific for individual bacteria, by correlating values obtained for tested individuals across different bacteria and Mathematicasoftware was used for drawing images. To analyze which subclass was dominant in the total IgG response, and which resemblesIgA response the most, reactivity of IgG, IgA, IgG1, IgG2, IgG3 and IgG4 of individual samples to a tested bacteria were mutually correlated, and highest correlation coefficient meant that the specific IgG subclass was the most dominant in the total IgG response.

## Results

### Specific anti-bacterial IgM

**Fig 1:**
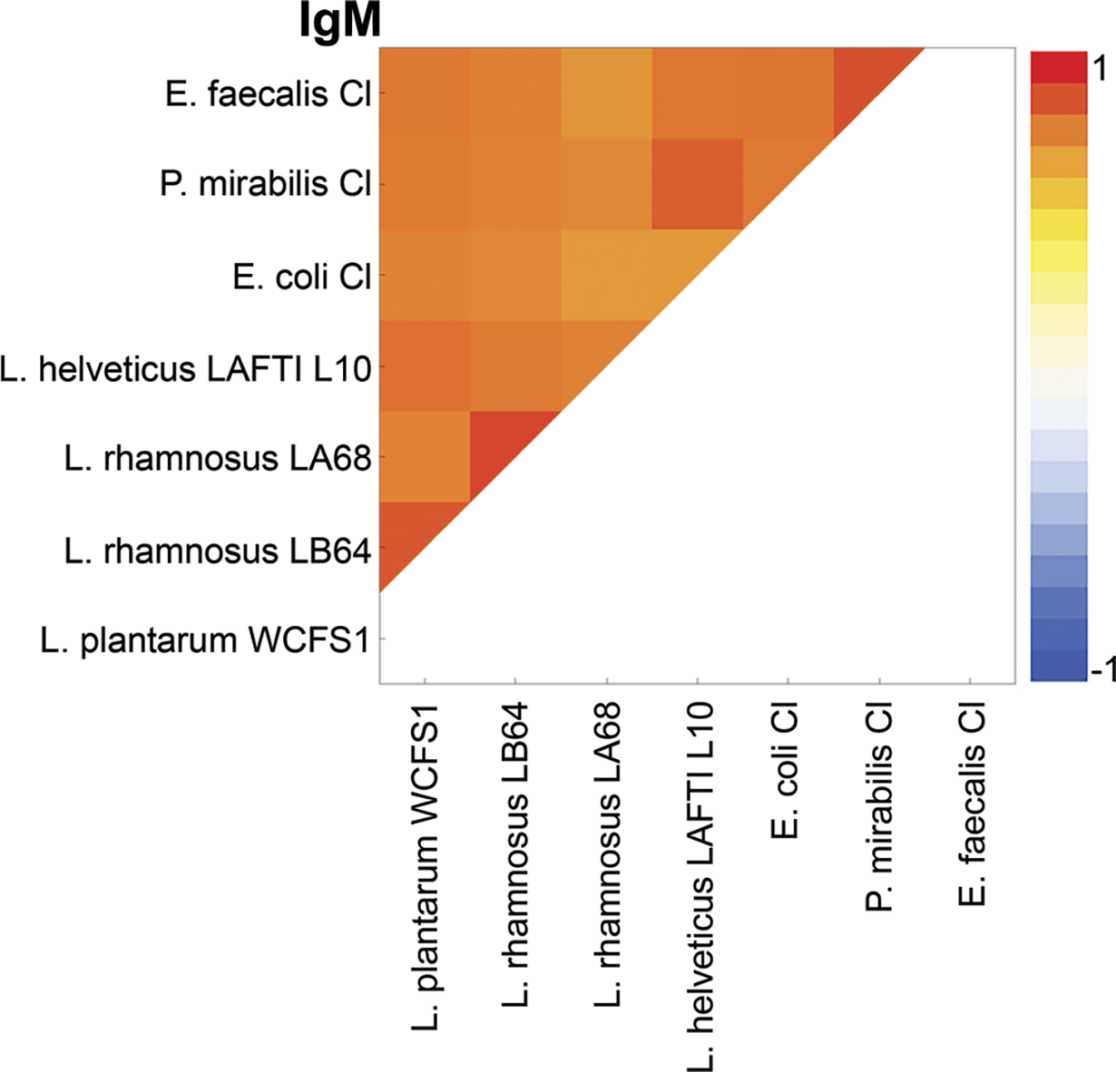
Graphic representation showing correlation coefficients obtained by correlating the string of reactivity of serum IgM from the tested population to different bacteria, obtained in ELISA. Red colour–Pearson product-moment correlation coefficient *r* = 1, Blue–*r* = -1.

The analysis of correlation coefficients of specific anti-bacterial IgM levels in different individuals, revealed highly positive correlation.Pearson product-moment correlation coefficientfor IgM fell between 0.7 and 0.92 (Fig 1), which indicated that IgM has broad reactivity, and was therefore not analyzed further.

### Specific anti-bacterial IgG

**Fig 2:**
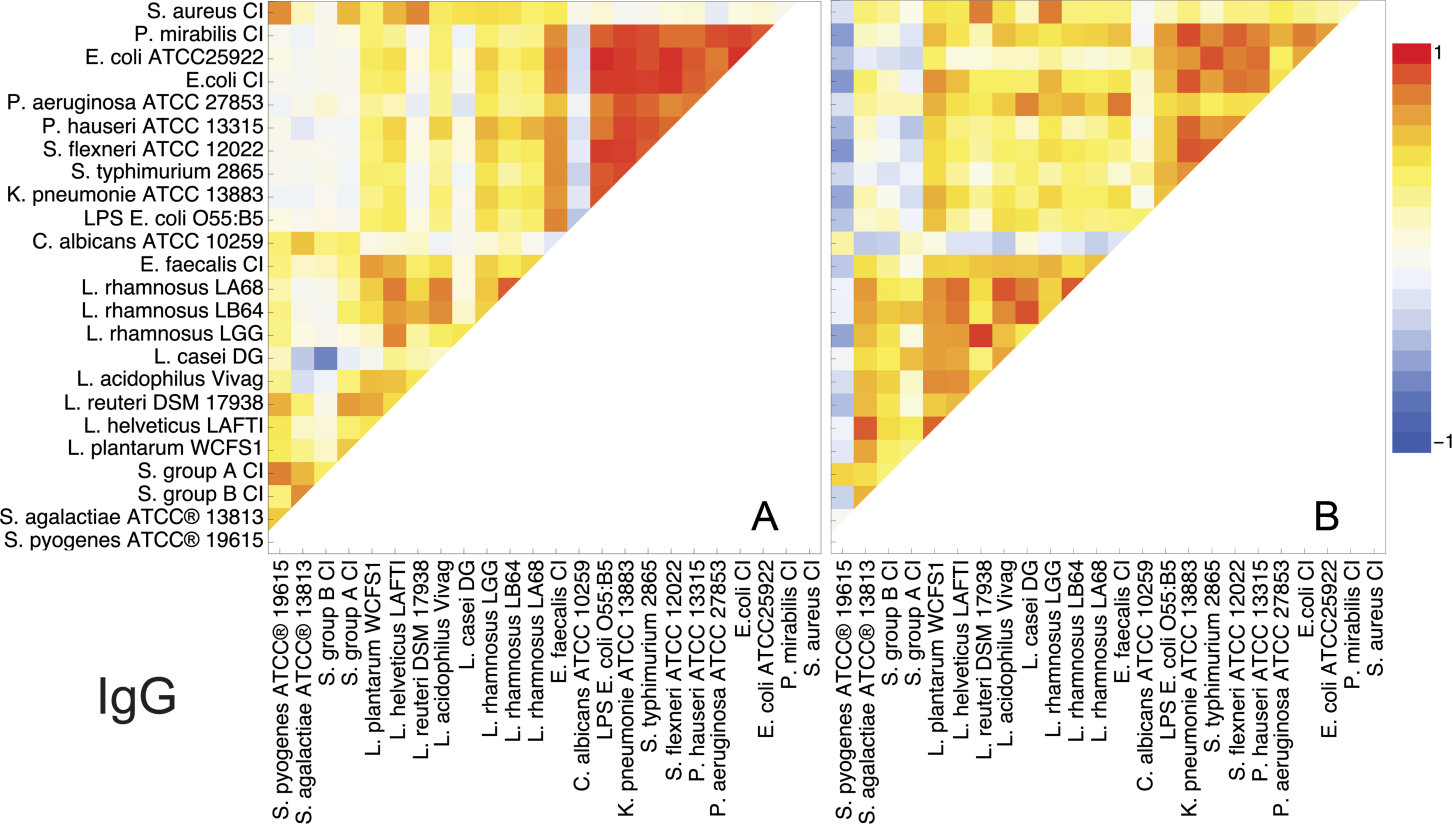
Graphic representation showing correlation coefficients obtained by correlating the string of reactivity of serum IgG ofdifferent individuals to different microorganism, obtained in ELISA, at steady state. A) Professional athletes; B) Control group. Red colour–Pearson product-moment correlation coefficient,*r* = 1, Blue–*r* = -1.

The analysis of correlation coefficients of specific anti-bacterial IgG levels in different individuals revealed a more complex and specific picture compared to IgM, as shown in Fig 2. Serum polyclonal IgG at a dilution of 1:400 clearly distinguishes Gram-positive (G+) and Gram-negative (G-) organisms, with *Candidaalbicans* being distinguished from all tested bacteria, except form *Streptococcus*,by close to zero correlation in both groups tested. No correlation in IgG levels was observed for *L*.*casei*, *L*. *reuteri*and *Streptococcus*withGammaproteobacteria in professional athletes, whereas for the control group there was also no correlation of different *Streptococcus* with Gammaproteobacteria, except for *S*. *pyogenes* which correlated negatively. Interestingly, *S*.*aureus* shows only modest similarity with other G+ (most *Lactobacillus* and *E*. *faecalis*) except with *L*. *reuteri* (0.77) and *S*. *pyogenes* (0.75); similar situation is also with the control group where *S*. *aureus*shows correlation with *L*. *reuteri* (0.79)and *L*. *rhamnosus* LGG (0.79). According to the IgG reactivity, evident from the Fig 2 is also a high positive correlation (0.76–0.98) found between G- in professional athletes, whereas this correlation is lower in the control group (0.39–0.91). Interestingly the opportunistic G+ bacteria *E*. *faecalis* CI showed a moderate/high correlation with G- bacteria in professional athletes, where this was not the case in the control group.

### Specific anti-bacterial IgA

**Fig 3:**
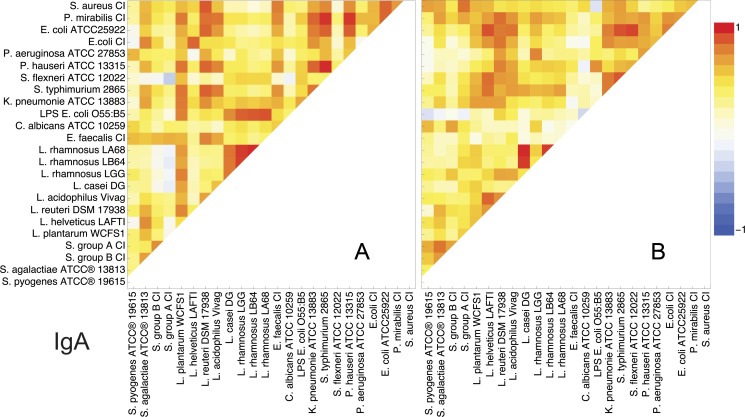
Graphic representation showing correlation coefficients obtained by correlating the string of reactivity of serum IgA of different individuals to different microorganism, obtained in ELISA, at steady state. A) Professional athletes; B) Control group. Red colour–Pearson product-moment correlation coefficient,*r* = 1, Blue–*r* = -1.

The correlation coefficient of IgA specific to different microorganisms/antigens yielded quite different result from IgG. Interestingly, only a weak positive correlation of LPS with G- bacteria was detected, in professional athletes, whereas in control individuals this was higher only for *P*. *houseri*(Fig 3). However, LPS showed an unexpectedly high r to the *L*. *rhamnosus* species,*L*. *plantarum* and *L*. *casei* DG in professional athletes which was absent in the control group. Overall, there are numerous similarities in IgA reactivity between the groups. Standing out is the absence of correlation in reactivity between LGG and other *L*. *rhamnosus* used in the control group.

**Fig 4:**
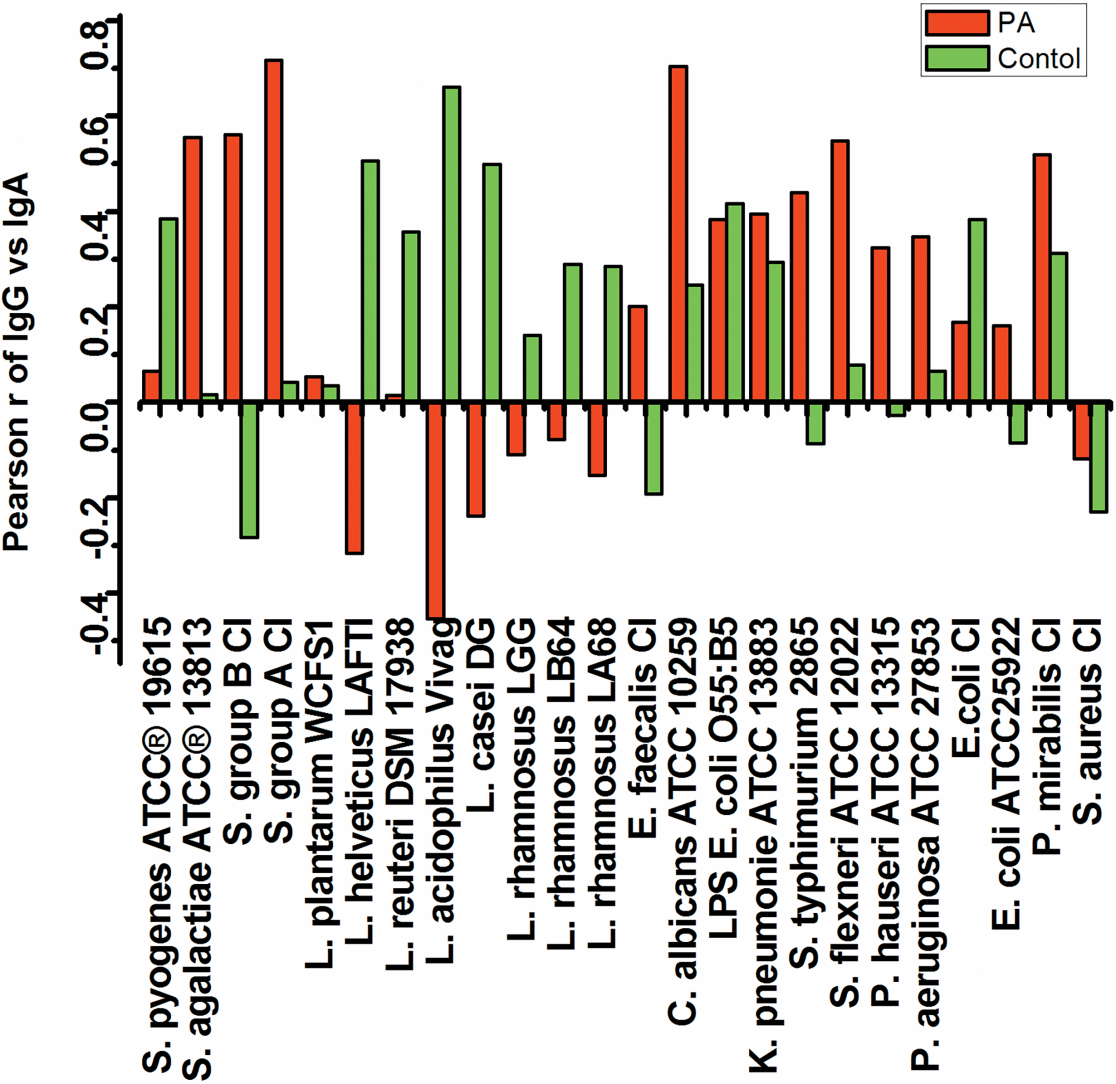
The strings of reactivities (apsorbance) of IgG and IgA to bacteria from different individuals were correlated, and the Pearson product-moment correlation coefficientswereplotted for individual bacteria. Red colour- Professional athletes PA; Green colour–Control.

Further, we looked into the correlation of IgG with IgA levels for different microorganisms in professional athletes, mainly to see if there are differences between probiotic and pathogenic strains, as a pathogen or opportunistic pathogen may induce both IgG and IgA responses. The analysis of IgA versus IgG indicated no or negative correlation for *Lactobacillus* species and *S*. *aureus* (-0.45 to 0.2), and no or positive correlation for Gammaproteobacteria, *E*.*faecalis*and *Streptococcus* species (0.15 to 0.55) (Fig 4). The strongest correlation (0.7) of IgG with IgA was observed for *C*. *albicans*and *Streptococcus* groupACI (Fig 4), showing that the subjects respond to these microorganisms by simultaneously producing both IgA and IgG antibodies. The results obtained for the control group were almost opposite (Fig 4), with lower correlation to *Streptococcus* and *Candida*, higher to *Lactobacillus*, and diverse correlation to Gammaproteobacteria.

### Prevalence of IgG subclasses

**Fig 5:**
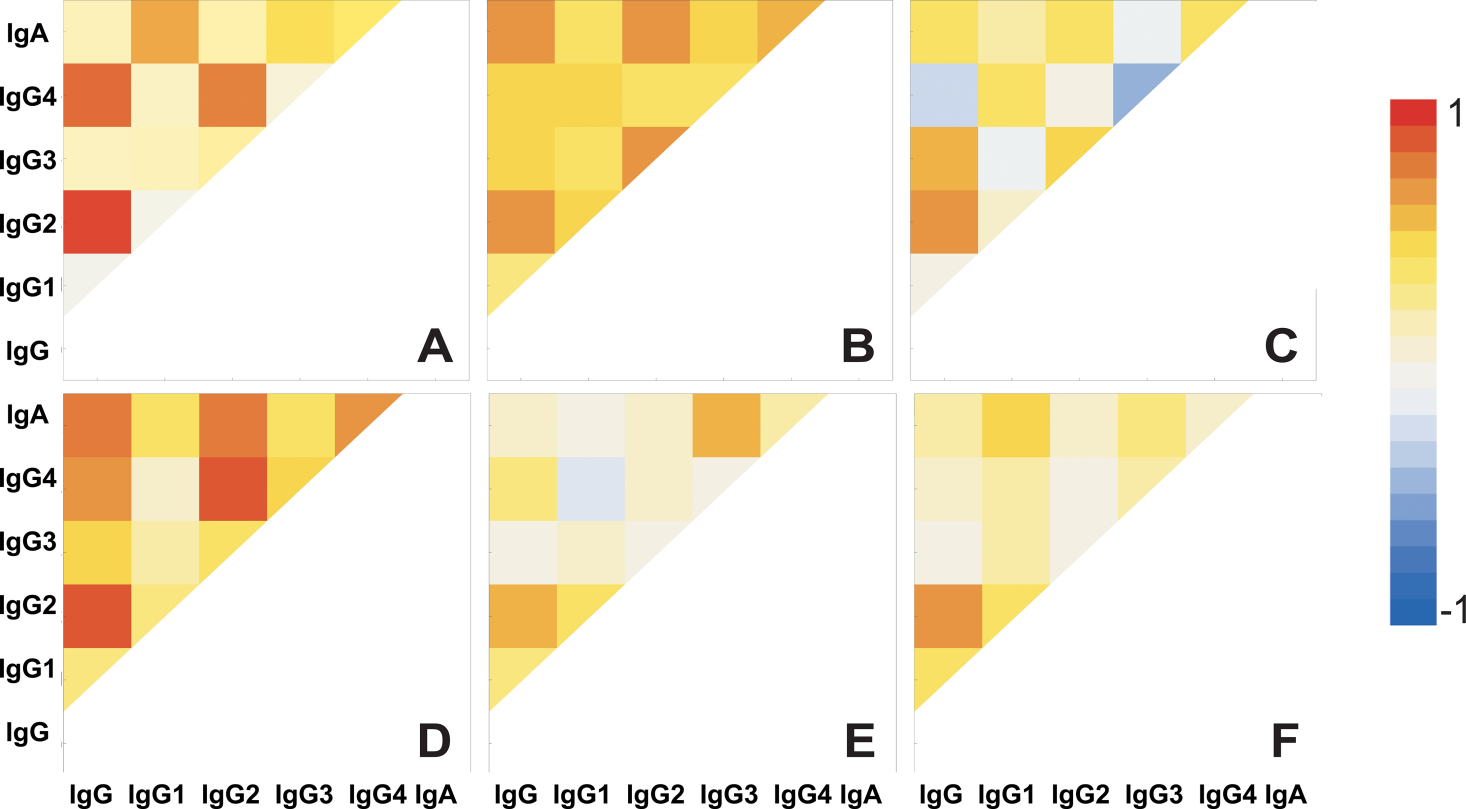
Graphic representation showing correlation coefficients obtained by correlating the string of reactivity of serumtotal IgG and total IgA with IgG subclasses, for different microorganisms in professional athletes. A) *Escherichia coli* ATCC25922; B) *Candida albicans* ATCC 10259; C) *Lactobacillus plantarum* WCFS1; D)*Salmonella typhimurium* 2865; E) *Lactobacillusrhamnosus* LGG, F) LPS from *E*. *coli*O55:B5.

Since total IgG reactivity is composed of different IgG subclasses reactivities, correlation coefficients were used to determine the most predominant IgG subclass in a specific response to a certain microorganism/antigen and reveal similarity of IgG subclasses with the IgA response (Fig 5A–5F). The IgG subclass level analysis was performed for six selected microorganisms/antigens. For LPS, *C*. *albicans*, and *E*. *coli*,*L*. *plantarum*, *L*. *rhamnosus* LGG, and *S*. *typhimurium* 2865 the total IgG showed highest correlation with IgG2.

### Titration of reactivity

**Fig 6:**
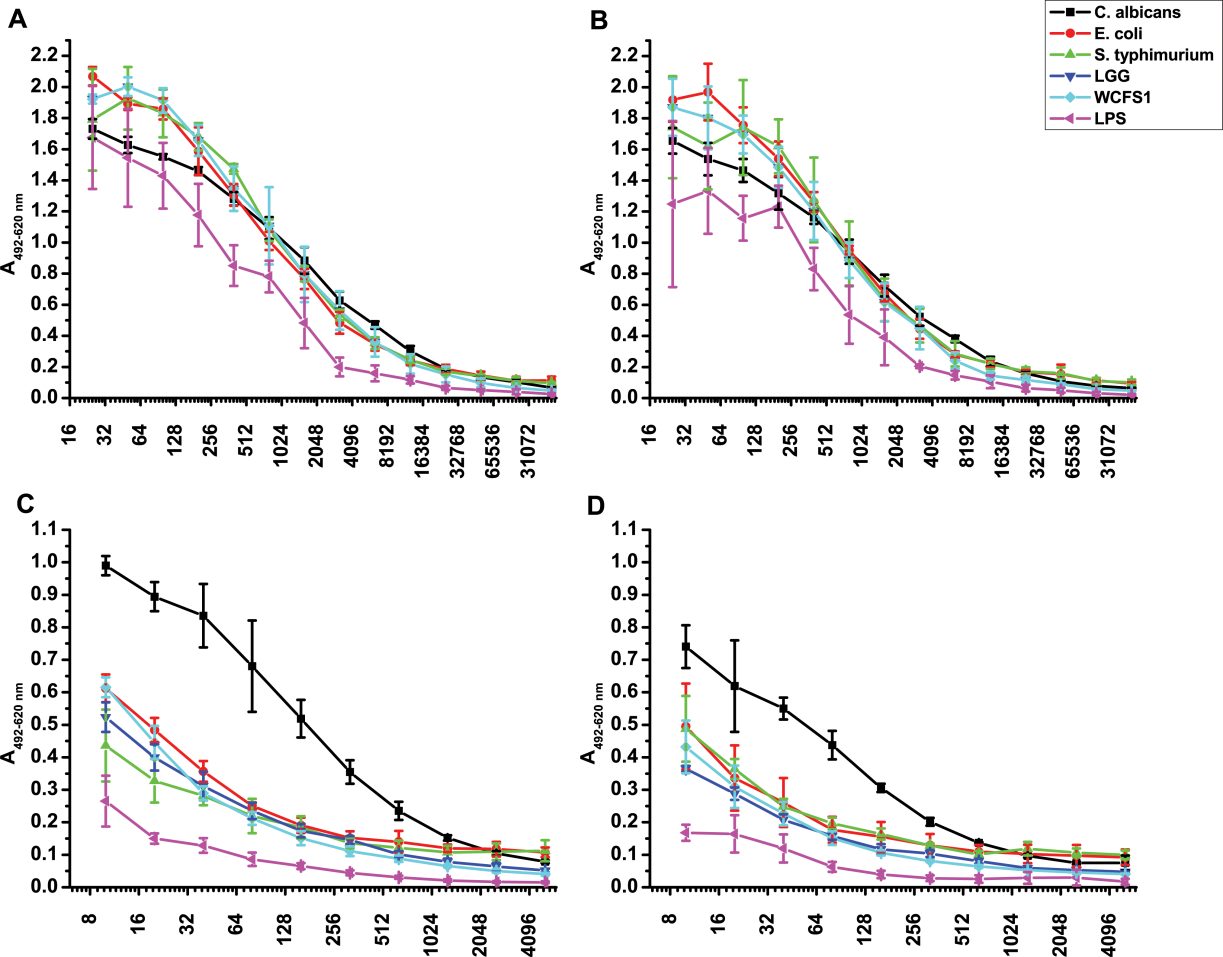
Analysis of reactivity of serial dilutions of serum IgG in A) professional athletes; B) control group, and of serum IgA in C) professional athletes; D) control group to different microorganisms. Red colour–*E*. *coli* ATCC25922; black -*C*. *albicans* ATCC 10259; turquoise–*L*. *plantarum* WCFS1; green–*S*. *typhimurium* 2865; blue—*L*. *rhamnosus* LGG, violet—LPS from *E*. *coli* 055:B5.

Titration of IgG and IgA reactivities wereperformed for the same microorganisms for which subclass analysis was performed. Curve fitting revealed that the obtained curves were not sigmoid in case of IgG, and in case of IgA the curve showed no saturation at lower dilutions (Fig 6). No substantial differences were found in the IgG reactivity towards the tested microorganisms, except in case of *C*. *albicans*, where significantly higher reactivity was found in professional athletes even at a dilution of 1:51200. Significantly higher were the reactivities of IgA from professional athletes at a dilution of 1:1280 for *C*. *albicans*, *L*. *plantarum* and *L*.*rhamnosus*. Also the curves for IgG were rather uniform for different microorganisms, with LPS showing less reactivity. In case of IgA *C*. *albicans* showed higher reactivity, and LPS lower.

## Discussion

In this study a direct ELISA was used as an only analytical assay, and as antigens whole microorganisms were attached to the plate, S1 Fig. Microorganism specific total IgG, and IgA levels were used for further comparison as IgM was shown to be uninformative as the most cross-reactive class (Fig 1), which is in accordance with the literature data and as IgM might be considered a part of innate immunity as natural antibodies [14].

In terms of similarity at the genus level it can be expected that Gammaproteobacteriawould show greater diversity. However, the obtained results show that there is very little difference in antibody selectivity among the tested species from the phylum *Proteobacteria*, which is especially obvious in professional athletes where the reactivity of IgG to LPS dominatesthe IgG reactivity to Gammaproteobacteria. Lipopolysaccharides consist of a hydrophobic domain known as lipid A (or endotoxin), a “core” oligosaccharide, and a distal polysaccharide (or O-antigen) [15]. Many G- bacteria, synthesize lipid A species [16], and yet variations of LPS are numerous. In *E*. *coli* more than 180 different O-antigens have been identified [17]. The mentioned differences in the LPS layer of G- bacteria are not reflected in the measurements of total serum IgG in professional athletes, whereas in the non athletic group, there is lower correlation of LPS with Gammaproteobacteria, than between individual Gammaproteobacteria.

Human plasma LPS originating from the G- bacteria and leaking through the mucosal surface of the GIT, has been recognized as the triggering factor for high-fat diet–induced diabetes and obesity [18].

Active training represents a good model to study limited inflammatory responses [11]. Namely, strenuous exercise induces high core body temperature and a reduction in splanchnic blood flow which leads to the disruption of tight junctions, which further leads to the increase of GI barrier permeability, bacterial translocation and higher levels of circulating endotoxins [19]. We would like to stress that we found no difference in titration curves between professional athletes and the control group towards LPS or the two G- bacteria tested. The differences could possibly be attributed to differences in affinity, which was not analyzed at this time.

On the other hand, IgG reactivity to the genus of probiotic *Lactobacillus* shows high diversity among different species, and even strains (Fig 2). This is probably due to the high diversity of the outer layer of *Lactobacillus*, the lack of a mutual immunodominant antigenic determinant or the presence of different kinds of blocking molecules, which prevent the binding to a common antigenic determinant, though certain grouping of reactivity is obvious in the control group pinpointing another difference between the groups.

What was puzzling for us was the capacity of IgA to distinguish between different geni among *Proteobacteria*, which was not the case for IgG in professional athletes (Fig 3), which would imply greater specificity of IgA, but it was previously found that the fraction of directly switched IgA (from IgM) represents approximately 90% of switched IgA cells in various immune compartments [20], which undermines the higher specificity of IgA. Also the titration curves show no saturation at very low dilutions, which tells of the lack specificity. In fact, as IgA is present on the frontline, at mucosal surfaces where risky interaction with microorganisms occur and may also be considered an innate immunity effector molecule [14], with increased avidity, like the IgM. The discrepancy might be related to the regulation of IgA production, namely as IgA class consists of subclasses IgA1 and IgA2 or to homing of IgA plasma cells. Salivary antibodies reacting primarily with cell wall carbohydrates, as well as dextrans (B1355 fraction S and B512) and phosphorylcholine are of the IgA1 subclass whereas IgA2 subclass dominates in the reactivity towards LPS and lipoteihoic acid [21], but this was not currently tested, as in this study only total serum IgA was analyzed.

The distinction between different antigens/microorganisms is also made by the predominant IgG subclass specific for the bacteria. It has long been known that human IgG responses to carbohydrate antigens, found on bacteria, are primarily restricted to the IgG2 subclass [22], which was also obtained here proving the concept of this analysis. Total serum IgG levels may vary considerably between healthy adults, but the proportion of each subclass is maintained within a relatively narrow range, by which the numeric designations of the subclasses were made: IgGl, 60–65%; IgG2, 20–25%; IgG3, 5–10%; IgG4, 3–6% [23], additionally each IgG subclass has an individual pattern of development with IgG1 and IgG3 attaining adult levels at an earlier age than IgG2 and IgG4, which is one of the reasons for higher susceptibility of infants to respiratory infections. Professional athletes experience moreupper respiratory tract infections (URTI) than recreationalathletes, with 30–70% ofillness episodes havingidentifiable pathogensassociated, of these bacterial infections represent a fraction [24].The most important result of this study is the high similarity of the tested G- LPS bearing bacteria as evident through IgG reactivity, and the dissonance in formation of IgG with IgA to probiotic strains, versus accordance for opportunistic pathogens in professional athletes, as well as the confirmation of IgG2 being the primary bacteria specific IgG subclass. Other interesting findings relate to the unexpected similarities noted between distant microorganisms especially in the control group IgG analysis. Professional athletes represent a specific population which is in a subclinical inflammatory state, with periodic increases in serum LPS levels upon strenuous performance. The presented experimental procedure is limited due to a small sample size, and a single dilution used for each antibody class, but this method of data analysis can be used for determining the similarities between microorganisms from the aspect of the humoral immune system, as well as for the analysis of different target populations. Both of the populations analysed here represented healthy young people who generally do not have increased antibody levels. This type of analysis could possibly be employed to identify key antigenic molecules of bacteria to which specific IgG is made in patients convalescing from bacterial infections, which could aid in formulating next generation of antibacterial monoclonal antibodies.

## Supporting information

S1 Fig *Lactobacillus rhamnosus* LA86 attached to a MaxiSorp ELISA plate. The attached bacteria were stained with Giemsa stain, the well was filled with distilled water and visualized under Inverted microscope, objective magnification 100x.
(TIF)

S1 Data Control reactivity to microorganisms, experimental data, absorbance. (XLSX)

S2 Data Professional athletes reactivity to microorganisms, experimental data. (XLSX)

S3 Data Titration, absorbances at serial dilutions. (XLSX)
