# Supplementary figures and images for: Profiling of microorganism-binding serum antibody specificities in professional athletes
Source: PLoS One. 2018 Sep 25;13(9):e0203665. doi: 10.1371/journal.pone.0203665 (PMC6155446; doi:10.1371/journal.pone.0203665)

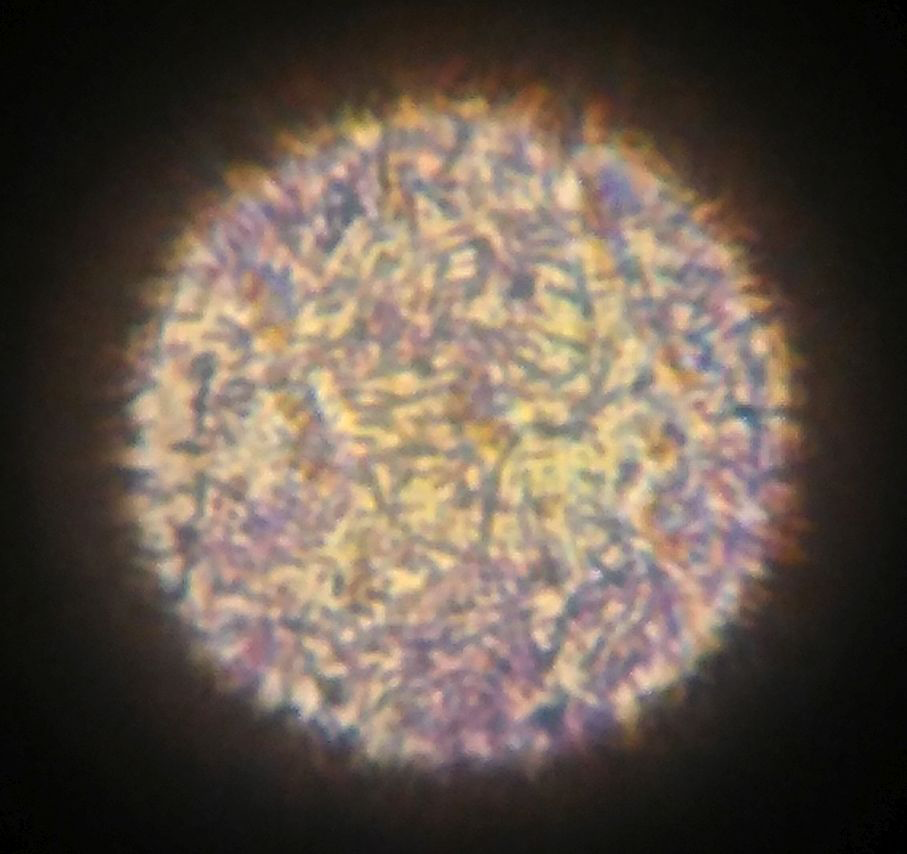

Supplement: S1 Fig — The attached bacteria were stained with Giemsa stain, the well was filled with distilled water and visualized under Inverted microscope, objective magnification 100x. (TIF) [file pone.0203665.s001.tif]
